# Stochasticity in plant cellular growth and patterning

**DOI:** 10.3389/fpls.2014.00420

**Published:** 2014-09-08

**Authors:** Heather M. Meyer, Adrienne H. K. Roeder

**Affiliations:** Department of Plant Biology, Weill Institute for Cell and Molecular Biology, Cornell UniversityIthaca, NY, USA

**Keywords:** variability, robustness, cell division, *Arabidopsis*, development

## Abstract

Plants, along with other multicellular organisms, have evolved specialized regulatory mechanisms to achieve proper tissue growth and morphogenesis. During development, growing tissues generate specialized cell types and complex patterns necessary for establishing the function of the organ. Tissue growth is a tightly regulated process that yields highly reproducible outcomes. Nevertheless, the underlying cellular and molecular behaviors are often stochastic. Thus, how does stochasticity, together with strict genetic regulation, give rise to reproducible tissue development? This review draws examples from plants as well as other systems to explore stochasticity in plant cell division, growth, and patterning. We conclude that stochasticity is often needed to create small differences between identical cells, which are amplified and stabilized by genetic and mechanical feedback loops to begin cell differentiation. These first few differentiating cells initiate traditional patterning mechanisms to ensure regular development.

## Introduction

During development, an organism acquires a characteristic shape and size and establishes robust organ morphologies. To achieve this, growing tissues undergo multiple rounds of growth, division, and differentiation until a highly organized structure is formed. For years, biologists have been fascinated with the idea of tightly regulated developmental processes that yield highly reproducible outcomes and have deemed seemingly random processes to be unimportant. However, recent evidence suggests that the underlying cellular and molecular mechanisms utilized to generate these reproducible structures often contain stochastic elements (Raser, 2005; Johnston and Desplan, 2010). Thus, there is a resurgent interest in randomness in biology.

Counterintuitively, stochasticity may be important for producing regular patterns. For instance, stochastic transitions between growth and disassembly phases of individual microtubules is critical for the rapid formation of ordered cortical microtubule arrays guiding the anisotropic expansion of plant cells (Allard et al., 2010; Eren et al., 2010). Dynamic instability allows the microtubules to explore various configurations and arrive at the optimal one (Holy and Leibler, 1994). Noise allows the disassembly of suboptimal configurations and thus allows the quick convergence on optimal ones.

**Figure 1:**
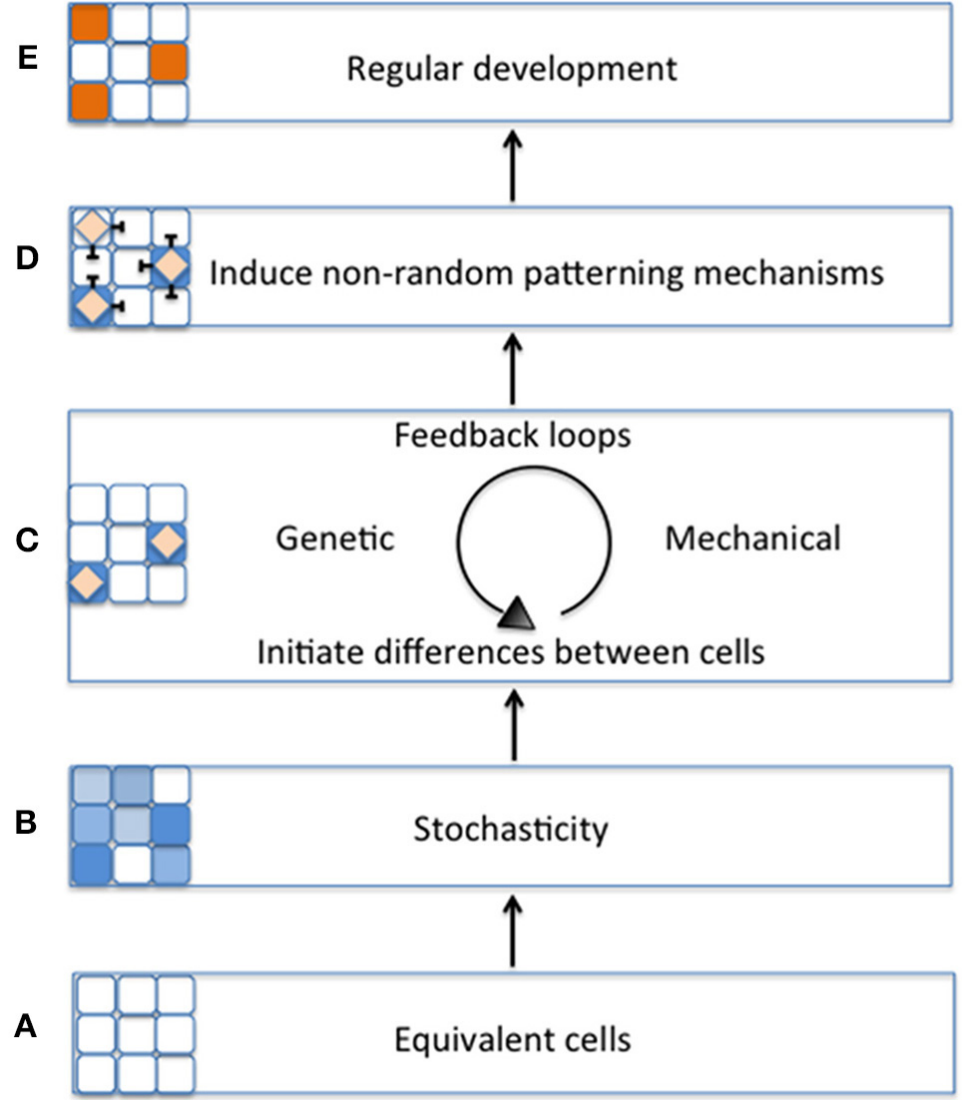
**Schematic model of the importance of stochasticity in promoting regular plant development. (A)** During early tissue development, cell start out as being morphologically equivalent (all white cells). **(B)** Equivalent cells exhibit initial differences from one another through stochastic fluctuations in gene expression (variation of blue cells). **(C)** Differences between cells will be stabilized by regulatory mechanisms such as genetic or mechanical feedback loops (blue cells with diamonds). **(D)** As the cell's fate is stabilized, it triggers non-random patterning mechanisms (e.g., lateral inhibition) **(E)** Patterning mechanisms promote regular tissue development (orange cells).

In various plant tissues, cells exhibit an immense amount of cell-cell variation. For instance, the *Arabidopsis thaliana* leaf epidermis is composed of various cell types, which range in cell size, shape, and DNA ploidy (Melaragno et al., 1993; Roeder et al., 2010; Elsner et al., 2012). Nonetheless, these tissues retain the correct organ morphology. Here we raise the question: does stochasticity at the cellular level contribute to reproducible tissue development in plants? In this review we examine how stochasticity is defined in biological systems and provide evidence that plants undergo stochasticity at the cellular level. Stochastic fluctuations of key regulators can initiate differences between equivalent cells. Genetic and mechanical feedback loops can enhance and solidify these differences to begin cell differentiation. Differentiating cells promote traditional patterning mechanisms, such as lateral inhibition, to further induce cell differentiation and patterning for proper tissue development (Figure 1). While in this review, our central focus is on regularity versus randomness in plant development, we draw many illustrative parallel examples from other systems with the intention of bringing further insight to the phenomenon of stochasticity in plants. For further discussions of the importance of stochasticity throughout plant development, please see the other reviews in this “Stochasticity in Plant Developmental Processes” research topic.

## What is stochasticity in a biological context?

*Stochasticity* is defined as “the quality of lacking any predictable order or plan” (TheFreeDictionary^1^) and has been long used to describe random or probabilistic events. For example, in the early 1900's Albert Einstein and Marian Smoluchowski described the zigzag behavior of Brownian particles (i.e., particles suspended in a fluid) as stochastic (Góra, 2006). Furthermore, fields such as mathematical finance use stochastic models to predict the behavior of financial markets (Malliavin and Thalmaier, 2006). More recently, stochasticity has been used to describe biological events, particularly noise in gene expression (Raser, 2005). How do we know what is stochastic, and how can we study stochasticity in a biological context?

Currently there are two major approaches for investigating stochasticity in biological systems. The first approach is to compare experimental results with those achieved through a stochastic computational model. If the model and experiments match, we can have some confidence that stochasticity plays a role in the process. The second approach is to test experimentally for differences in the behaviors of two identical systems due to stochastic noise. The difficulty with this approach is to be sure that the systems are truly identical. Therefore, this approach has been used primarily to study stochasticity of gene expression in single cells.

**Figure 2:**
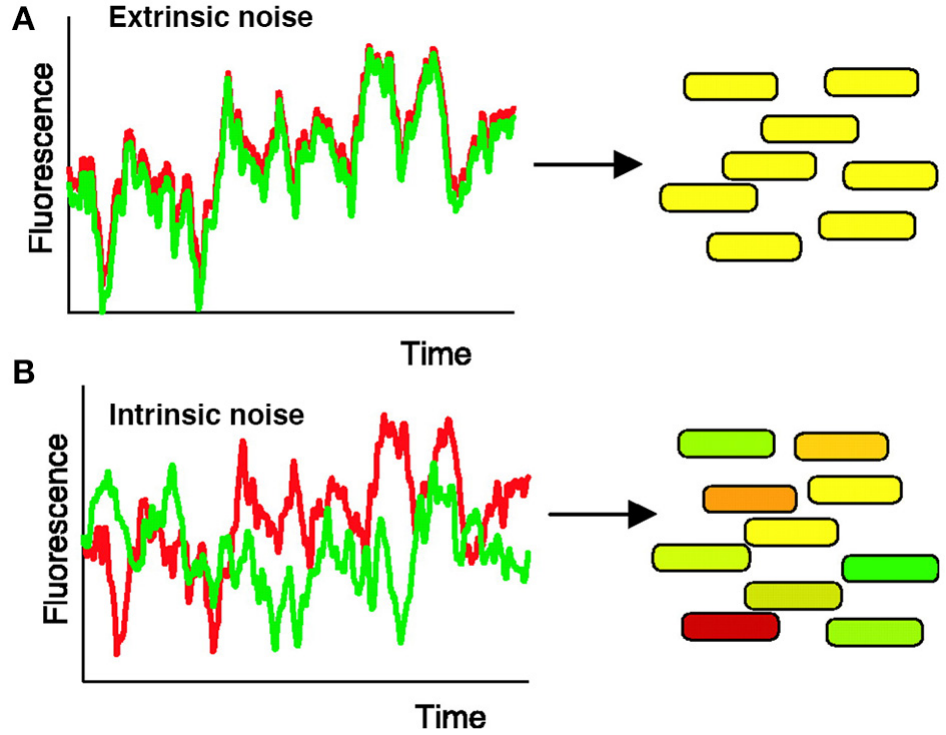
**Measuring intrinsic and extrinsic noise in *E. coli***. Intrinsic noise can be differentiated from extrinsic noise by measuring the activity of two allelic GFP variants, YFP (shown in red) and CFP (shown in green), under control of the same promoter. **(A)** When intrinsic noise is absent within a single cell, the fluorescence intensity of YFP and CFP should be identical (represented by yellow cells). Extrinsic noise will cause different cells within the population to exhibit variations in overall fluorescent intensity. **(B)** Intrinsic noise within a single cell will cause the fluorescence intensity of YFP and CFP to differ, resulting in some green and some red tinted cells. Reproduced from Elowitz et al. (2002). Reprinted with permission from AAAS.

For instance, Elowitz et al. (2002) tested how stochastic gene expression influences cellular variability in *Escherichia coli*. To do this, Elowitz et al. constructed a strain of *E. coli* in which two fluorescent alleles (cyan and yellow) are integrated into equivalent chromosomal loci under the control of the same promoter (Figure 2). Elowitz et al. subsequently analyzed fluorescent intensities of these reporters using fluorescence microscopy and computerized image analysis. Using these analyses, they found differences in expression between the cyan and yellow alleles within a single cell, indicating the presence of intrinsic noise, noise caused by the inherent randomness in transcription and translation of a particular gene (Figure 2B). Furthermore, they found variation in the overall fluorescent intensity between cells, suggesting the presence of extrinsic noise, noise attributed to fluctuations in environment (Figure 2A).

The results from these experiments suggest that both intrinsic and extrinsic noise in gene expression have a significant impact on promoting phenotypic diversity. For example, in *Bacillus subtilis* noise in the genetic network allows a few cells to stochastically and transiently become competent to take up extracellular DNA in response to stress while most other cells sporulate (Süel et al., 2006). By creating a diversity of cellular responses the survival of the population is optimized. Many have used this dual reporter system to demonstrate how stochastic gene expression influences phenotypic diversity in single-cell systems (Elowitz et al., 2002; Ozbudak et al., 2002; Rao et al., 2002; Blake et al., 2003; Raser and O'Shea, 2004; Pedraza and van Oudenaarden, 2005; Raser, 2005; Rosenfeld, 2005; Bar-Even et al., 2006; Newman et al., 2006).

Multicellular organisms may utilize stochastic mechanisms similar to those seen in unicellular organisms. However, the biological techniques necessary to assess how stochasticity contributes to cell behavior within a multicellular system are limited. Inventing new techniques to biologically assess and manipulate stochasticity will be important to advance our understanding of the role of randomness in biology in the future.

## Stochasticity in plant cell growth and division

Plants undergo continuous development throughout their lifespan and thus have specialized mechanisms to carefully control organ size and structure even when exposed to extreme environmental conditions. However, despite the regularity observed on an organ level, plant cells exhibit a high degree of variability in growth and division, suggesting that stochasticity is present and even maintained in tissues.

**Figure 3:**
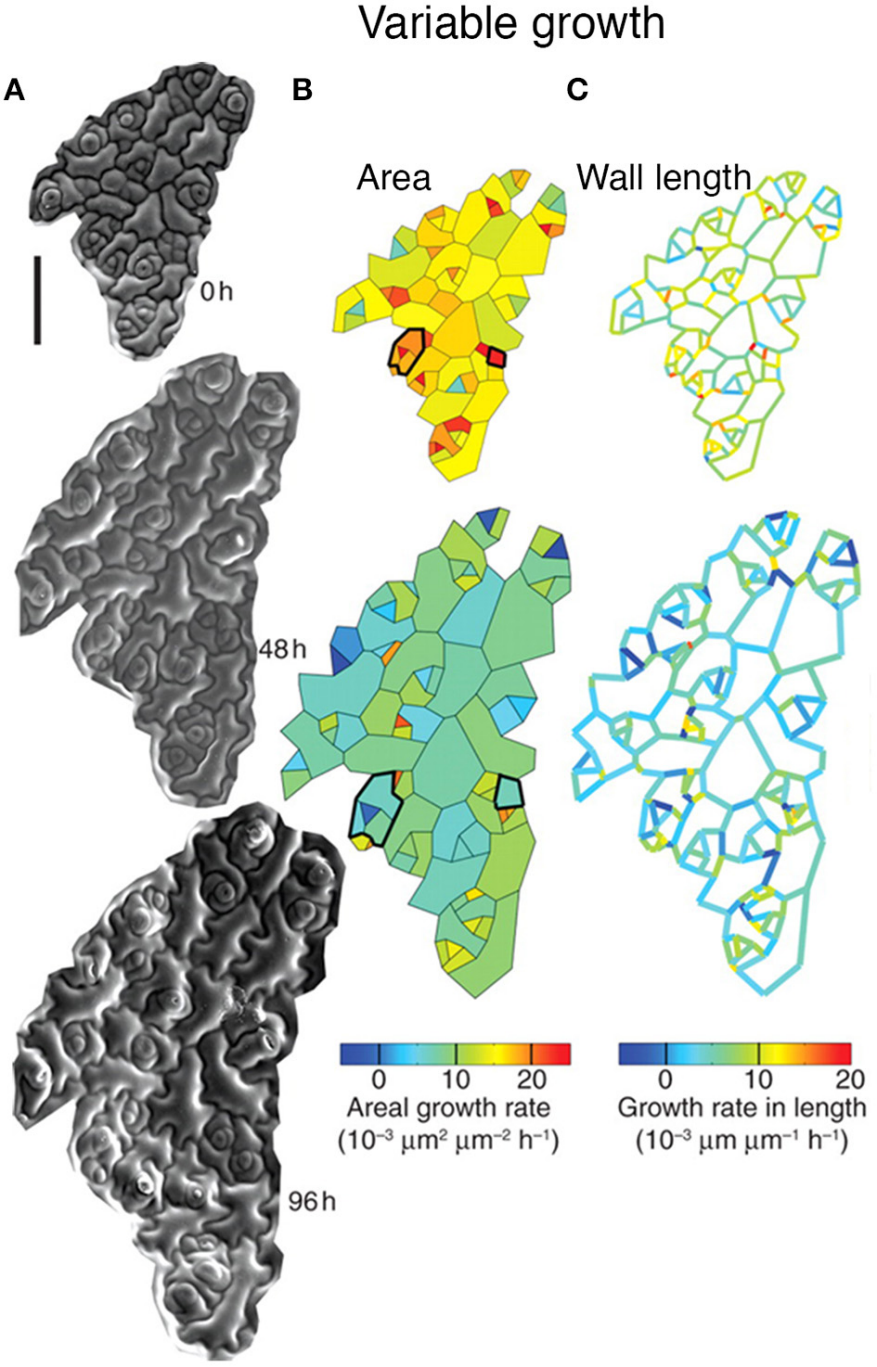
**Leaf epidermal cells exhibit growth variability**. Cellular growth heterogeneity is apparent from **(A)** serial scanning electron micrographs (SEMs) of a growing *Arabidopsis* leaf over 96 h. The analysis columns show the **(B)** areal growth rate and **(C)** growth rate in length. Cells that change their areal growth rate in time are outlined in black. Reprinted from Elsner et al. (2012) with permission from Oxford University Press.

Elsner et al. (2012) have found considerable variability in the growth rate of individual cells in the *Arabidopsis thaliana* leaf epidermis. Using time-lapse imaging and statistical analysis, they demonstrate that neighboring cells have dramatically different growth rates (Figure 3). Remarkably, individual walls of the same cell often have different growth rates (Figure 3C). In addition, cells were found to be highly dynamic, often changing their growth rates over time (Figure 3B, outlined in black). Elsner et al. found no correlation between growth rate and cell size, nuclear size, or anisotropy, suggesting that there is no obvious external cause for differences in growth rate. Similarly to leaves, cell growth variability has been observed in the plant meristem, suggesting that growth variability may be a general trend (Kierzkowski et al., 2012; Uyttewaal et al., 2012). Is cell growth variability due to stochasticity, or has the source for promoting variability not yet been identified? The answer requires further investigation.

**Figure 4:**
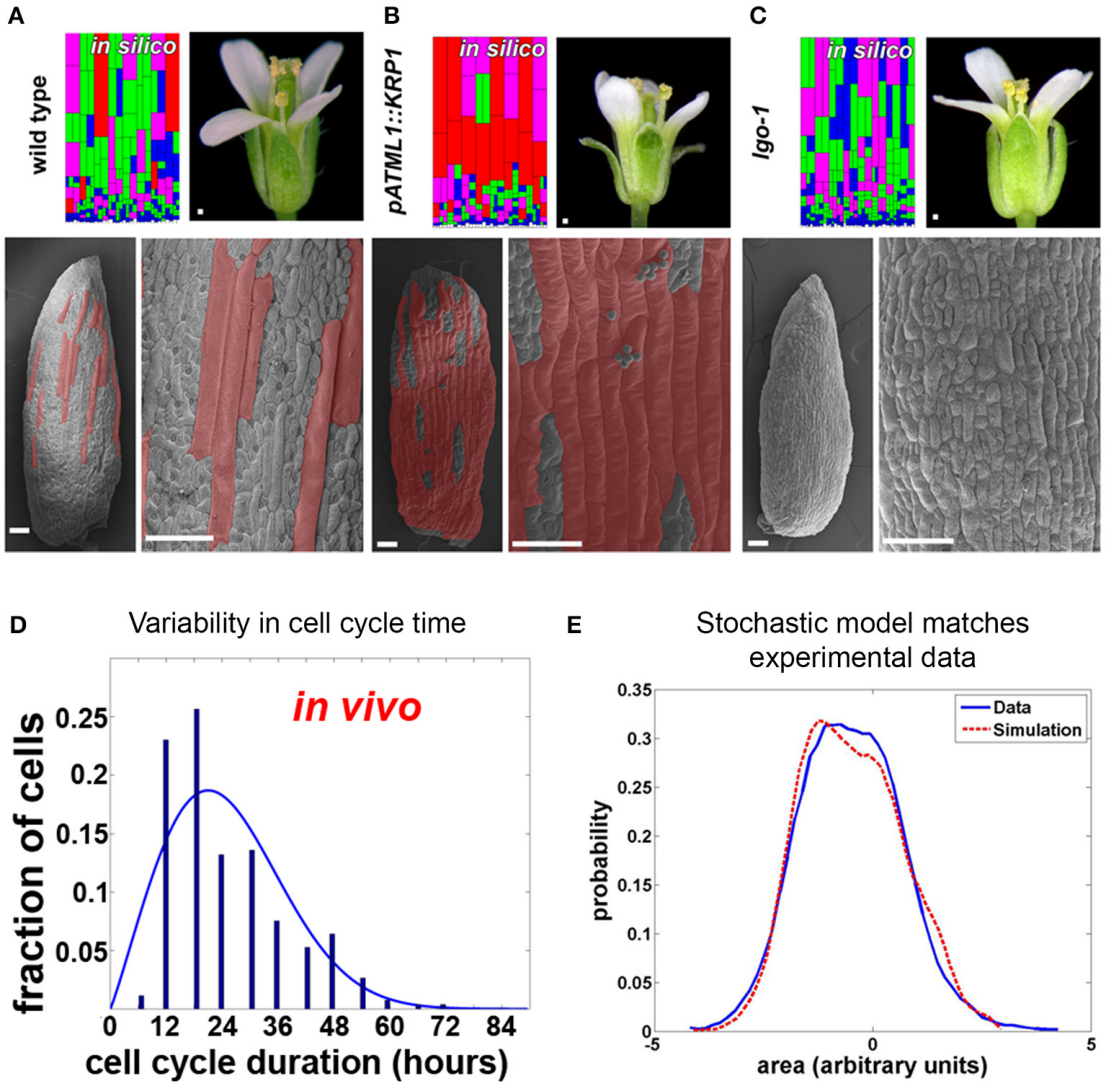
**Timing of *Arabidopsis* sepal epidermal cell division and entry into endoreduplication is variable. (A)** Wild type sepals (the outermost, green, floral organs) have a diversity of cell sizes on the outer epidermis ranging from giant cells (false colored red in SEMs) to small cells (not colored). A stochastic computational model that accounts for cell cycle length and entry into endoreduplication can accurately predict the distribution of sepal cell sizes from wild-type plants. In the model, cells are colored according to the number of endocycles they have undergone (red = 3, purple = 2, green = 1, and blue = 0). **(B,C)** Changing the expression of cyclin dependent kinase inhibitors (CKI) alters the probability of entry into endoreduplication and the resulting cell size distribution. As the model predicts, **(B)** plants overexpressing cyclin-dependent kinase inhibitor *KRP1* form ectopic giant cells, but islands of small cells remain between giant cells. Note that the sepals curve outward. **(C)** Likewise the model predicts that plants mutant for cyclin-dependent kinase inhibitor *LGO* continue to have variable smaller cell sizes. Note that the sepals curve slightly inward. **(D)** The duration of the cell cycle amongst sepal cells is highly variable and ranges from 12 to more than 60 h. **(E)** A histogram of the cell sizes produced in the random model (red dashed line) is not significantly different from measured cell sizes in the sepal (blue line). This figure is modified and reprinted from Roeder et al. (2010) under the Creative Commons Attribution license.

Substantial variability in the timing of cell division has been observed in the *Arabidopsis* sepal epidermis (Roeder et al., 2010). Live imaging of sepal development and tracking of cell lineages have revealed two sources of variability. First, in dividing cells, the length of the cell cycle ranged from about 12 h to more than 60 h (Figure 4D). Second, differences were found in the time at which cells stop dividing (i.e., exit the mitotic cell cycle) and enter endoreduplication, a cell cycle in which cells grow and replicate their DNA but bypass division (i.e., become polyploid). As a consequence, mature sepals contain cells with a range of ploidies from 2 to 16C. A similar pattern of cells with ploidy varying from 2 to 16C is present in the leaf epidermis, suggesting that variability in the timing of endoreduplication occurs broadly (Melaragno et al., 1993). The cells continue to grow while they endoreduplicate, such that the 16C cells become giant, stretching about one-fifth the length of the sepal. Giant cells are distributed between smaller cells with a wide range of sizes and ploidies (Figure 4A).

Are cell cycle length and timing of entry into endoreduplication stochastic in the sepal epidermis? A stochastic computational model can reproduce the *in vivo* sepal cell size distribution, which reflects both cell cycle length and endoreduplication state, indicating that stochasticity is a plausible scenario (Figures 4A,E; Roeder et al., 2010). In the model, cells make random decisions shaped by probability distributions that reflect the experimental data about cell cycle length and endoreduplication timing (Figure 4D).

The model was further tested to determine whether it could reproduce the effects of biological perturbations in the cell cycle (Roeder et al., 2010). Altering the expression of cell cycle regulators, such as cyclin-dependent kinase inhibitors, was shown to change the probability of endoreduplication, indicating that stochasticity can be genetically regulated. When cyclin-dependent kinase inhibitors were overexpressed, the number of highly endoreduplicated giant cells found on the sepal epidermis increased; however, the outcome was still probabilistic, as not all cells endoreduplicated (Figure 4B). Changing the probability distribution in the model replicated the mutant (Figure 4B model), suggesting that the underlying decision is still stochastic. Conversely, in a cyclin-dependent kinase inhibitor mutant, the most highly endoreduplicated giant cells are absent, but the remaining cells still exhibit considerable variability (Figure 4C). Again, the phenotype of the mutant could best be modeled as a shift in the probability distribution of the timing of endoreduplication (Figure 4C model). Further, the finding that the epidermal specification pathway in *Arabidopsis* promotes this variability in sepal cell size suggests that developmental regulators sometimes promote stochasticity in cell behavior (Roeder et al., 2012). Interestingly, overall sepal organ size does not significantly change when mutations that alter a cell's ability to endoreduplicate are introduced (Roeder et al., 2010). How organ morphology is robust to the stochasticity in cell size requires future investigation.

In addition to cell growth and division, the orientation of the new cell wall is somewhat variable. Recently, both Dupuy et al. (2010) and Besson and Dumais (2011) have used mathematical modeling to predict the probability with which a cell will divide along a given plane for a variety of plant cells. Their models suggest that the probability with which a cell chooses a given division plane is related to differences in the geometry of the choices. In the 1880s, Errera suggested that the new cell wall takes on the configuration that minimizes its surface area. If two possible planes of cell division have nearly equal surface areas, the cell will adopt each plane with nearly equal frequency (Besson and Dumais, 2011). If one possible plane of cell division has a much lower surface area than the second possibility, the first plane has a much higher probability of being chosen. Besson and Dumais demonstrated that plant cells do in fact divide probabilistically as predicted: the second, third, and even fourth most optimal division planes can be observed. Complementary to those results, it has recently been demonstrated that auxin transcriptional response is important in breaking the default geometric rule to promote asymmetric cell divisions in the developing embryo (Yoshida et al., 2014). This suggests that genetic components can influence the probability that a cell will adopt a particular division plane.

The examples presented above imply that plants utilize stochastic mechanisms throughout their lives. Nevertheless, even when cell growth, division and orientation are altered, a plant still has the ability to create organs with robust morphology. Thus, does stochasticity in cellular development really matter? We next look to examples from other systems to see how regularity can emerge from stochasticity and assess the implications of these studies for plant development.

## Stochasticity can produce regular patterns during development

Frequently during development, cells differentiate and form highly organized patterns to determine the finalized function of a tissue. Recent studies are now finding that noisy cellular processes often initiate these robust developmental processes. Thus, how does a tissue reach the same reproducible developmental outcomes when the events leading to them are stochastic? Here we will discuss examples in which stochasticity has been demonstrated to facilitate reproducible phenotypic outcomes.

### Stochasticity promotes cellular phenotypic heterogeneity

Stochasticity can be instrumental in producing regular development through initiating a diversity of cell types from a uniform population of cells. For example, cancer cell populations often exhibit subpopulations of different phenotypic cell types, where the proportions of each subpopulation remain remarkably constant over time. The ability for cancer cells to maintain this cell-state equilibrium has been the subject of immense research and debate for many years, but recent studies counterintuitively implicate stochasticity in the maintenance of equilibrium (Gupta et al., 2011; Wang et al., 2014). For example, in human breast cancer cell populations, cells trend toward equilibrium among three morphologically distinct cell subtypes: stem cell-like, luminal, and basal, where luminal or basal cells make up the majority of the population and stem cell-like cells make up the minority (Fillmore and Kuperwasser, 2008; Mannello, 2013). Although stem cell-like cells only constitute a small percentage of total cancer cell population, researchers have assumed that they are likely to be the most important cell type because of their ability to self-renew and differentiate into the other cancer cell types (Mannello, 2013).

**Figure 5:**
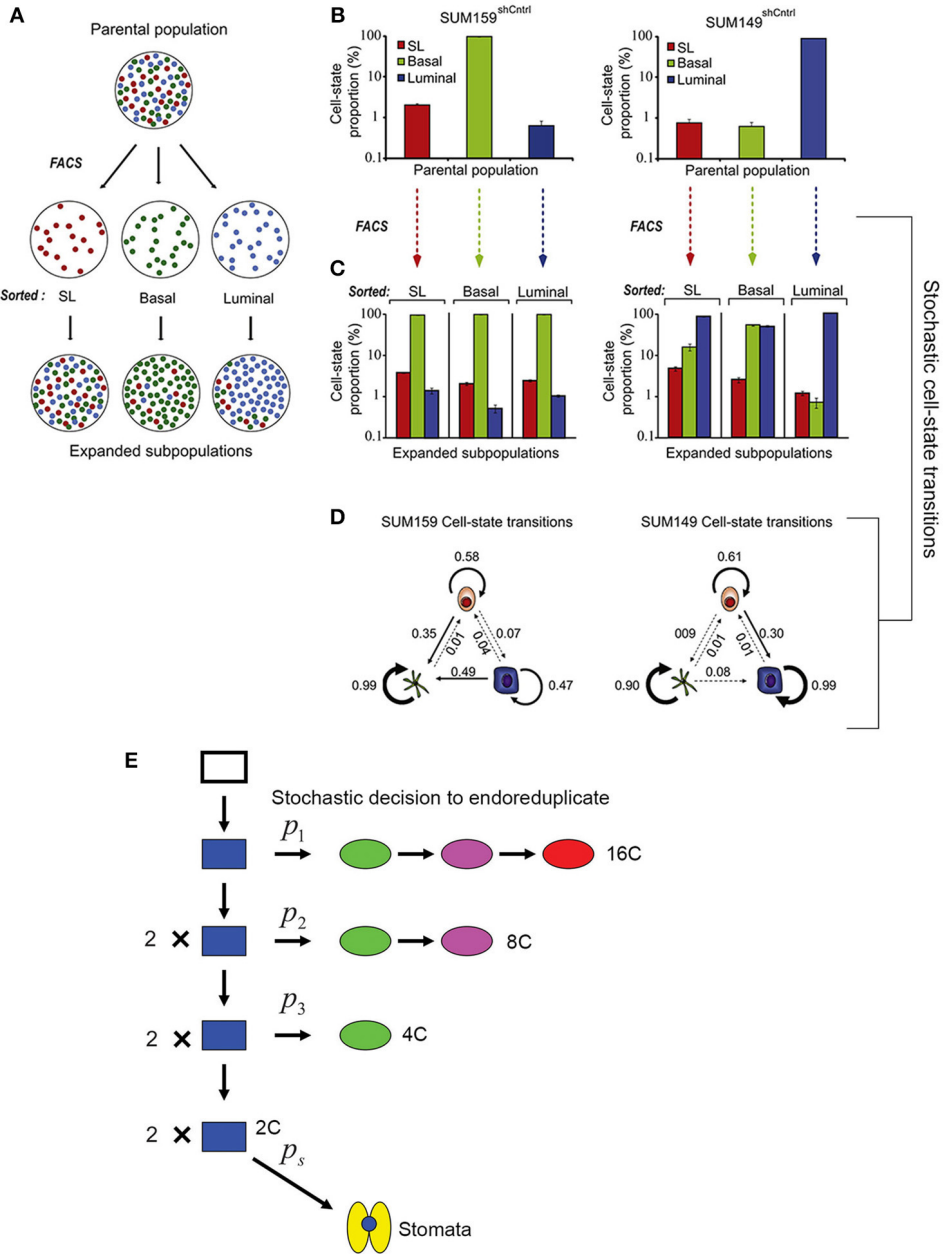
**Stochasticity maintains phenotypic diversity in cell populations. (A)** Cancer cell subpopulations [stem cell-like cells (SL; red), basal (green), and luminal (blue)] were sorted using fluorescence-activated cell sorting (FACS) to isolate cell type specific populations. After 6 days post-sorting, each of the isolated populations expanded to contain all three cell subtypes, suggesting that all three cell types can interconvert stochastically between different cell states. **(B)** Graph showing the proportion of SL, basal, and luminal cells in two cancer cell lines before sorting. **(C)** Cells were FACS sorted to produce populations with a single pure cell type. The graph shows the that 6 days after sorting the proportion of SL, basal, and luminal cells in each of these populations has returned nearly to the starting proportions. **(D)** Schematic diagrams depicting the stochastic cell state transitions between cell types for each cell line. Reprinted from Cell, 146/4, Gupta et al. (2011) Copyright, with permission from Elsevier. **(E)** A probabilistic model for the endoreduplication of a population of *Arabidopsis* epidermal cells. A 2C cell (blue) may either randomly decide with the probability p1 to either enter endoreduplication or mitotically divide. If the cell enters endoreduplication in the first cell cycle, it will continue to endoreduplicate until it becomes a 16C giant cell (red). If the cell undergoes mitotic division, then each daughter cell has a random probability p2 of entering endoreduplication. If a cell enters endoreduplication in the second cell cycle, then it will continue to endoreduplicate until it becomes an 8C cell (purple), whereas if it mitotically divides, then each daughter cell will once again have a random probability p3 of entering endoreduplication or dividing. If in the third cell cycle a cell enters endoreduplication, then it will become a 4C cell (green), whereas if it doesn't it will remain a 2C (blue) cell. Those final 2C cells have the probability ps to become a stomatal cell (yellow). This figure is reprinted from Roeder et al. (2010) under the Creative Commons Attribution license.

Gupta et al. (2011) demonstrated that stochastic processes are necessary to promote breast cancer cell-state equilibrium. Using fluorescence-activated cell sorting, Gupta et al. purified stem cell-like, basal, and luminal cells from two breast cancer cell lines (SUM149 and SUM159) and saw that after 6 days, these isolated cell types regenerated populations containing all three cell types in their original proportions, thus returning to a state of equilibrium (Figures 5A–C). Given the behavior of these cells, Gupta et al. proposed that cells undergo stochastic interconversions between different cell-states. Using a Markov-based approach—which assumes that the probability for a cell to transition is based solely on its current state and not its previous state—they modeled probabilistic transitions between cancer cell subtypes (Figure 5D).

Through experiments Gupta et al. show that in returning to equilibrium, cultured cancer cells and mouse models actually undergo the cell state transitions that are predicted by the stochastic model, some of which are unexpected. For example, the model predicts that approximately 1 day after sorting, a sharp spike of luminal differentiation will occur from stem cell-like SUM159 cells. As predicted, this spike is observed experimentally. The most insightful prediction of the model is that luminal and basal cells can de-differentiate back into cancer stem cells, a process observed in the sorted populations. These results imply that killing cancer stem cell-like cells is not an effective strategy because differentiated cells may revert back into stem cell-like cells. Thus, stochasticity is not only needed to maintain phenotypic diversity in cancer cells but may also serve as a valuable mechanism for maintaining phenotypic equilibrium during development.

Based on this study, we might similarly hypothesize that stochasticity within plant cell division is necessary to maintain cell size diversity in the epidermis. Similar to the cancer model, transitions in sepal cell state have been modeled probabilistically (Figure 5E; Roeder et al., 2010), but it should be noted that sepal epidermal cells are unable to interconvert or revert once a cell size has been established. Despite the variability, sepals typically have similar proportions of cells in each size class, and having the correct proportions of these cells is necessary for the organ's final function. Forming the correct number of highly endoreduplicated giant cells is critical for controlling the curvature of the sepal (Figure 4A). *Arabidopsis* sepals curve inward to protect the developing flower bud and then straighten to allow the flower to bloom. In plants in which the cell cycle has been altered to increase the number of giant cells, the sepals curve excessively outwards (Figure 4B) (Roeder et al., 2010, 2012). However, when there are no giant cells, the sepals curve excessively inwards (Figure 4C). Thus, although the overall size of the sepal is not affected by changes in the distribution of cell size, the curvature is, which likely affects the sepals' ability to protect the developing floral organs. Thus, we see that stochasticity can be important for regular development through ensuring a diversity of cell types. Next, we consider the mechanism through which stocasticity can initiate this cellular diversity and its regulation to generate patterns.

### Patterning mechanisms are initiated through stochasticity

Biologists oftentimes regard cell fate determination in well-defined systems as being highly regulated. For example, during multicellular development, a cell must acquire a specific cell fate in contrast to its neighbor to create cell patterns in order to define a tissue's final structure and function. One common patterning mechanism is lateral inhibition, in which one cell sends out an inhibitory signal to prevent its neighbors from adopting the same identity. Although lateral inhibition has been well-studied, the mechanism by which it is initiated is still a mystery. However, increasing evidence suggests that stochasticity is required to initiate many of these lateral-inhibition patterning mechanisms, one of which has been explored experimentally in Notch-Delta signaling.

**Figure 6:**
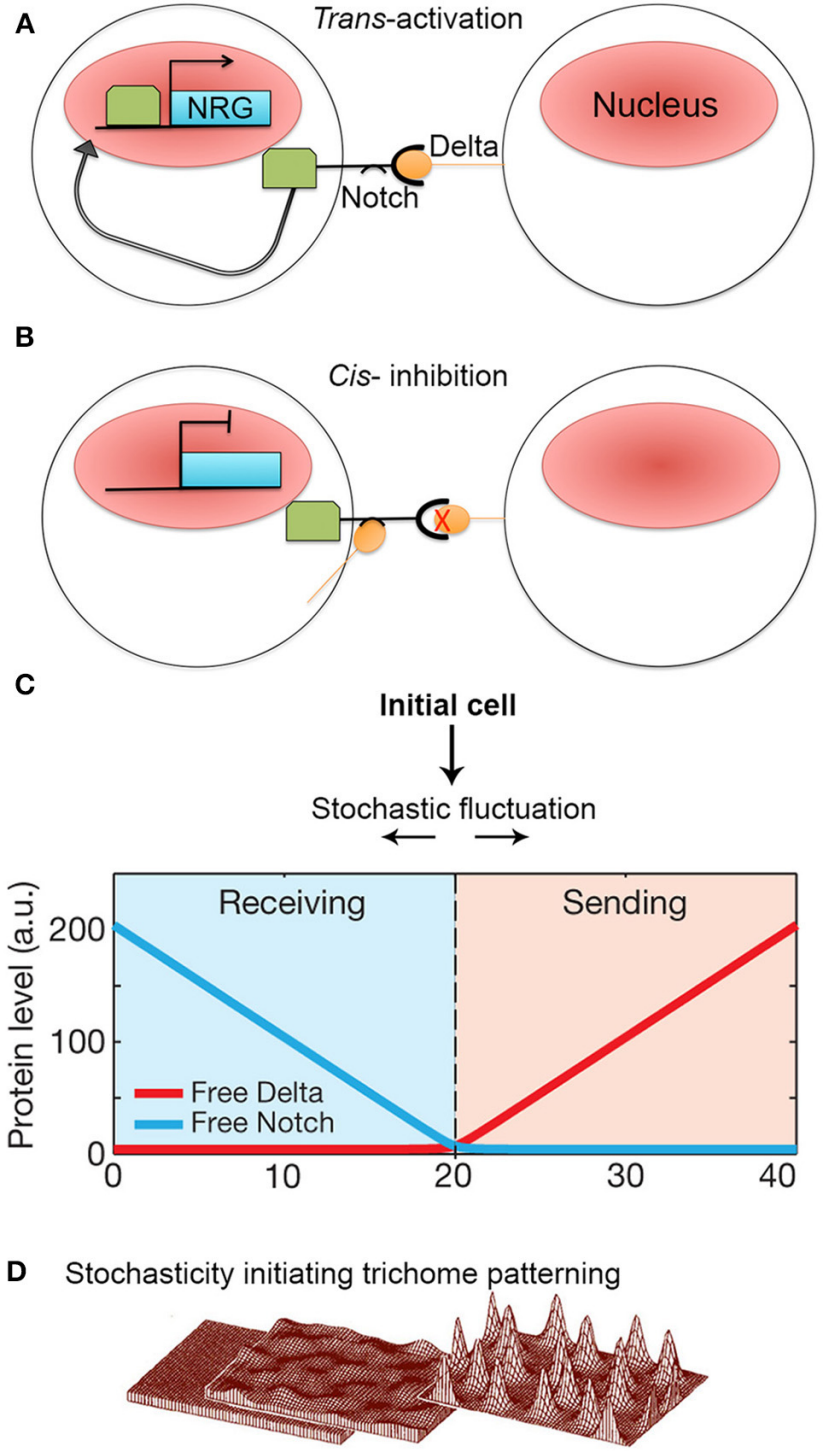
**Stochasticity initiates lateral inhibition-based patterning. (A)** A simplified model of the Notch-Delta pathway. When transmembrane receptor Notch on one cell interacts with ligand Delta on the neighboring cell, Notch is cleaved and intracellular domain subsequently moves to the nucleus to *trans*-activate Notch-responsive genes (NRG). **(B)** However, if Delta interacts with Notch in the same cell, Notch is inhibited and unable to activate NRGs. Additionally, Notch becomes less responsive to *trans*-Delta. **(C)** When Notch and Delta are present in the same cell, they mutually inactivate one another. This creates a “switching” environment, where small concentration imbalances between Notch and Delta due to stochastic fluctuations will either cause a cell to transition to a sending state (high delta, low notch) or a receiving state (low delta, high notch) thus activating the patterning process. Reprinted with permission from MacMillan Publishers Ltd: Sprinzak et al. (2010). **(D)** Computational models of *de novo* trichome patterning based on Meinhardt's model, which suggests that trichome patterning is initiated via small stochastic changes in activator and inhibitor concentrations in equivalent cells (left to middle). Regulatory positive feedback loops to amplify small concentration differences. In addition inhibitor factors prevent surrounding cells from differentiating into trichomes producing a spaced pattern (middle to right). Reprinted by permission from MacMillan Publishers Ltd: Hülskamp (2004).

The Notch-Delta signaling pathway is an excellent model of lateral inhibition in animals, where it induces cell pattering in a variety of development processes, including neural differentiation in *Drosophila* and vertebrates (Morrison et al., 2000; Wakamatsu et al., 2000; Kubu et al., 2002). Notch is a transmembrane receptor protein. Activation occurs when Notch, which resides on the membrane of one cell, interacts with a Delta ligand that resides on the neighboring cell (i.e., *trans*-activation). This activation causes proteolytic cleavage of the intracellular Notch domain, which translocates into the nucleus and acts as a transcription factor to activate Notch-responsive genes (Figure 6A; del Álamo et al., 2011).

The Delta ligand has additionally been implicated in inhibiting Notch activity within its own cell (i.e., *cis*-inhibition) (Figure 6B). *Cis* Notch-Delta interactions are thought to mutually inhibit one another, where Notch inactivates expression of Delta and vice versa. This inhibitory interaction is assumed to make a cell less receptive to receiving *trans*-Delta signals from its neighboring cells (del Álamo et al., 2011). The ability for Notch to be *trans*-activated and *cis*-inhibited by Delta suggests that a cell's responsiveness to Delta signaling depends on Notch-Delta concentration ratios. During patterning, how does an imbalance in Notch and Delta expression occur between identical neighboring cells such that one cell expresses more Delta whereas the other cell expresses more Notch?

To explore this question, Sprinzak et al. (2010) investigated the transcriptional response of Notch when subjected to different levels of either *cis* or *trans*-Delta in cell culture. The authors observed that when Notch is exposed only to varying levels of *trans*-Delta, Notch responds in a graded manner. Conversely, when Notch is exposed to a strong pulse of *cis*-Delta, Notch is inactivated. As the *cis*-Delta concentrations become diluted over time, Notch remains inactivated until *cis*-Delta reaches a particular dilution threshold, which then prompts a sharp spike of Notch activation.

These observations were modeled with the major assumption that Notch and *cis*-Delta mutually inhibit each other. In this model, Sprinzak et al. found that mutual inactivation between Notch and Delta induces a sensitive “switching” environment. In other words, small stochastic concentration changes of either Notch or Delta will lead to a cell being in a “sending state” (i.e., a cell exhibits high Delta concentrations and low Notch concentrations) or a “receiving state” (i.e., high Notch concentrations but low Delta concentrations) (Figure 6C). The validity of this model was tested through simulations that mimicked cellular wing-vein boundaries in *Drosophila*. These simulations not only successfully recapitulate the developmental processes seen in wild type, but also the phenotypes seen Notch and Delta mutants. This model demonstrates that a small amount of random noise is sufficient to induce a genetic switch, causing one cell to become different from its neighbor. Once a cell adopts either a sending or receiving state, typical Notch-Delta lateral inhibition intercellular signaling propagates throughout the tissue to produce a regular pattern of spaced specialized cells. Thus, regular patterning could not occur without stochastic fluctuations in Notch and Delta to initiate the process (Figure 1D).

Although Notch-Delta signaling is not present in plants, lateral inhibition is an important patterning mechanism, particularly in the spacing of trichomes (hair cells) on leaves and non-root hair cell files in the root (Hülskamp and Schnittger, 1998; Lee and Schiefelbein, 2002; Schellmann et al., 2002; Scheres, 2002; Schiefelbein, 2003). The mechanism of trichome spacing is thought to follow an Alan Turing reaction-diffusion model (Meinhardt and Gierer, 1974; Hülskamp, 2004; Benítez et al., 2007; Morelli et al., 2012). According to the model, during leaf development, equivalent cells will start to express low levels of trichome-specific transcriptional activators. The activators up-regulate their own expression forming a positive feedback loop. These activators also activate a set of rapidly diffusing inhibitors, which will move to the neighboring cells and inhibit expression of the activators. Since all cells initially start with equal concentrations of activators and inhibitors, stochastic fluctuations in activator concentration are required to initiate the autoregulatory feedback loop in a few cells that leads to the accumulation of activators and the specification of trichome identity (Figure 6D; Benítez et al., 2008; Bouyer et al., 2008; Dupuy et al., 2008). Simultaneously, the inhibitors diffuse rapidly to accumulate in surrounding cells blocking trichome identity in the neighbors. Thus—similar to lateral inhibition with Notch-Delta signaling—stochasticity in the levels of regulatory proteins is required to initiate the patterning mechanisms that break the symmetry of identical cells and to allow neighboring cells to adopt different identities.

## Regulation of stochastic mechanisms

While stochasticity is often needed to initialize cellular decisions, regulatory mechanisms are often utilized to stabilize those decisions. For instance, in *Saccharomyces cerevisiae*, yeast cells must determine a single bud site in order to produce a new daughter cell. Normally, this occurs via the accumulation of polarity-regulating factors, such as GTP-Cdc42p and its effectors, at specific locations marked by immobile landmark proteins (e.g., Rsr1) (Bender and Pringle, 1989; Toenjes et al., 2004). Remarkably, in the absence of landmark proteins, yeast still select a single site for bud polarization and emergence, but the selection occurs at random (Irazoqui et al., 2003; Wedlich-Soldner et al., 2003).

In this case, regulatory feedback loops are needed to ensure that only one bud site is chosen. Initial studies using mathematical modeling suggest that accumulation of polarity complexes occurs via a positive feedback loop that eventually leads to determining the polarization axis (Kozubowski et al., 2008; Howell et al., 2009). However, these models do not fully explain the observed behavior of polarity factors. Oftentimes, polarity complexes are observed accumulating and re-locating at multiple sites before a final site is picked. Thus, it is hypothesized that polarity regulator complexes compete with one another until only one complex is victorious (Goryachev and Pokhilko, 2008; Howell et al., 2009).

**Figure 7:**
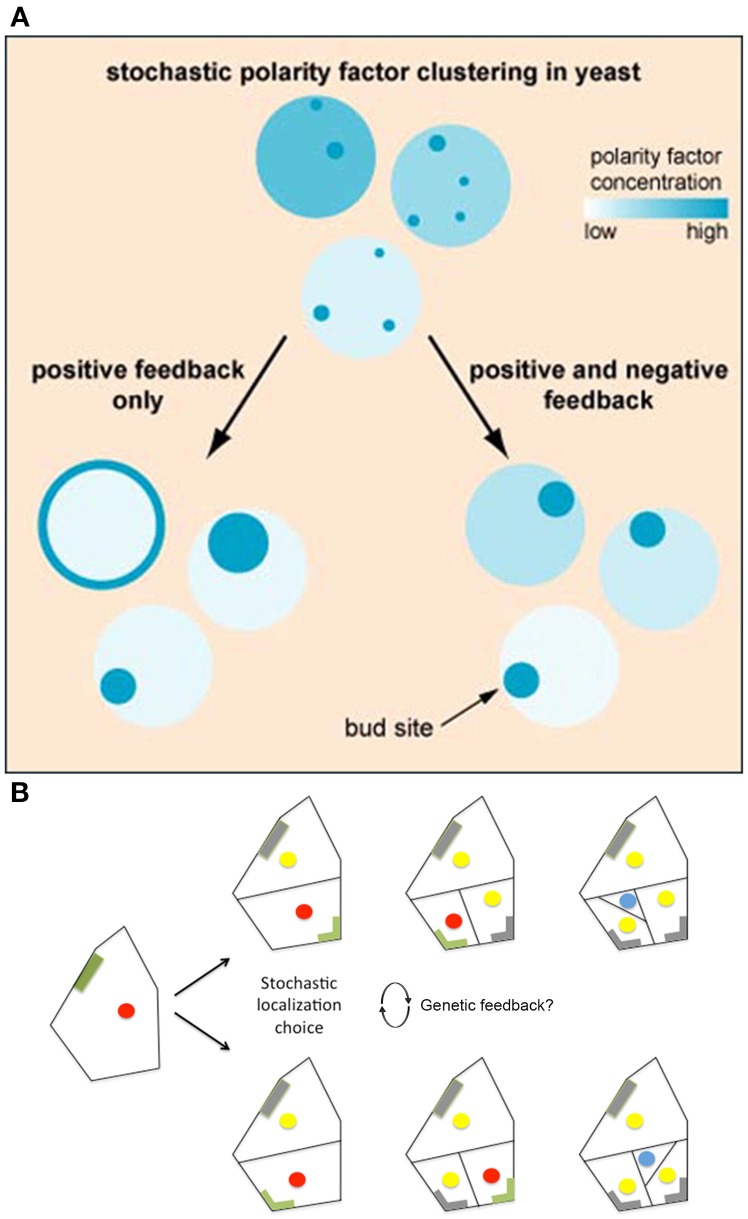
**Regulatory mechanisms stabilize cellular decisions initiated by stochasticity. (A)** Yeast must select a single bug site and when landmark proteins are absent the site is selected stochastically. Computational modeling suggests that both positive and negative feedback loops are necessary to amplify and yet constrain this random bud polarization to ensure that a single bud site is selected. In models with only positive feedback loops, stochastic fluctuations in polarity regulating complexes increase, causing polarity regulators to either disperse on the plasma membrane or form large patches (left). However, when negative feedback is added to the positive feedback model, a cell becomes less susceptible to noise, causing proper polarization (right). Reprinted from Howell et al. (2012) Copyright, with permission from Elsevier. **(B)** BASL undergoes polarity switching during asymmetric cell division in *Arabidopsis*. BASL (green) accumulates on the cell periphery farthest from the intended division plane. Once asymmetric division occurs, BASL disappears (gray) and re-accumulates on the farthest cell periphery of the newest daughter cell. When two equivalent locations in the periphery of the cell are available for BASL accumulation, BASL appears to pick one randomly. This raises the question of whether BASL accumulation is regulated by a similar stochastic mechanism to that observed in bud site selection in yeast. The blue dot represents the formation of a guard mother cell, the red dot represents nuclei with BASL accumulation, and the yellow dot represents nuclei without BASL accumulation. Adapted from Robinson et al. (2011). Reprinted with permission from AAAS.

Howell et al. (2012) used high-resolution filming, modeling, and genetic analyses to investigate the formation of polarity-regulating complexes *in vivo*. Howell et al. found that in some yeast cells, not only do multiple polarity complexes form and compete with one another, but also that concentrations of these complexes oscillate, suggesting that these factors may be regulated via a negative feedback loop. To test this hypothesis, Howell et al. developed a series of models that tested the consequences of various feedback loops on polarity. They saw that when models only incorporated a positive feedback loop, stochastic concentrations of polarity regulators were amplified, making cells very sensitive to protein concentration changes and often causing regulators to disperse throughout the plasma membrane (Figure 7A). Whereas, if negative feedbacks were added to the positive feedback in the model, the polarization was more robust because the negative feedback decreased a cell's sensitivity to stochastic concentration changes (Figure 7A). This suggests that even if a cell employs stochastic mechanisms, the overall noise may be partially filtered through feedback loops (Figure 1C). Filtering stochasticity via genetic regulation has also been observed in other biological systems. For example, during *Caenorhabditis elegans* development, *skn-1* normally buffers stochasticity in the intestinal specification gene network. Mutations in *skn-1* cause downstream genes to become susceptible to random fluctuations of expression, which consequently generates phenotypic variation during intestinal cell differentiation (Raj et al., 2010).

Conversely, models of phyllotaxy (i.e., the spacing pattern of primodia emerging from the meristem) emphasize how robust patterns can emerge from cellular interactions that retain a high level of stochasticity (Jeune and Barabé, 2006; Jönsson et al., 2006; Smith et al., 2006; Stoma et al., 2008; Mirabet et al., 2012). However, careful analyses of phyllotactic patterning reveal that noise at the local level can have visible consequences at the whole-organism scale. For instance, fluctuations in the temporal sequence of organ initiation can cause permutations in the sequence of primordia initiations, leading to new phyllotactic angles between successive siliques along the stem (Mirabet et al., 2012; Besnard et al., 2014). Altogether, this suggests that stochasticity is not simply buffered out, but plays a regulatory role in pattern formation in both plants and animals.

In plants, an analogous example of cell polarity generation is BASL-mediated asymmetric division in stomatal patterning. BASL polarizes as a crescent in the periphery of the cell located opposite the position where the next asymmetric stomatal lineage division will occur (Dong et al., 2009). BASL also localizes dynamically to the nucleus (Dong et al., 2009).

Interestingly, in stomata lineage cells, BASL has been shown to undergo polarity switching to establish the orientation of the asymmetric cell divisions in a similar manner to Cdc24—one of the polarity regulating proteins needed for yeast bud site selection. In stomatal precursor cells, BASL is initially localized in the nucleus and the cell periphery farthest from the future site of the asymmetric division plane (Dong et al., 2009; Robinson et al., 2011). Once asymmetric division occurs, BASL will re-localize once again to the cell periphery farthest from this new cell wall, switching polarity post-mitosis (Figure 7B; Dong et al., 2009; Robinson et al., 2011). In a few instances, however, stomatal precursor cells have symmetric geometry with respect to the new cell wall, in which case BASL has equal probability of localizing on either side of the cell (Figure 7B; Robinson et al., 2011). In this case, only one polarity site is chosen, and it has been suggested that small stochastic fluctuations of BASL are instrumental in the random choice of wall.

How BASL localizes and undergoes polarity switching is currently unknown. However, deletion variant studies have shown that the C-terminal region of BASL is needed for peripheral cell wall localization and is sufficient to rescue the *basl-1* phenotype (Dong et al., 2009). As more information about BASL is revealed, it will be interesting to see whether it is subjected to regulated stochasticity as observed in yeast bud site selection.

### Stochastic processes are modulated via mechanical properties

**Figure 8:**
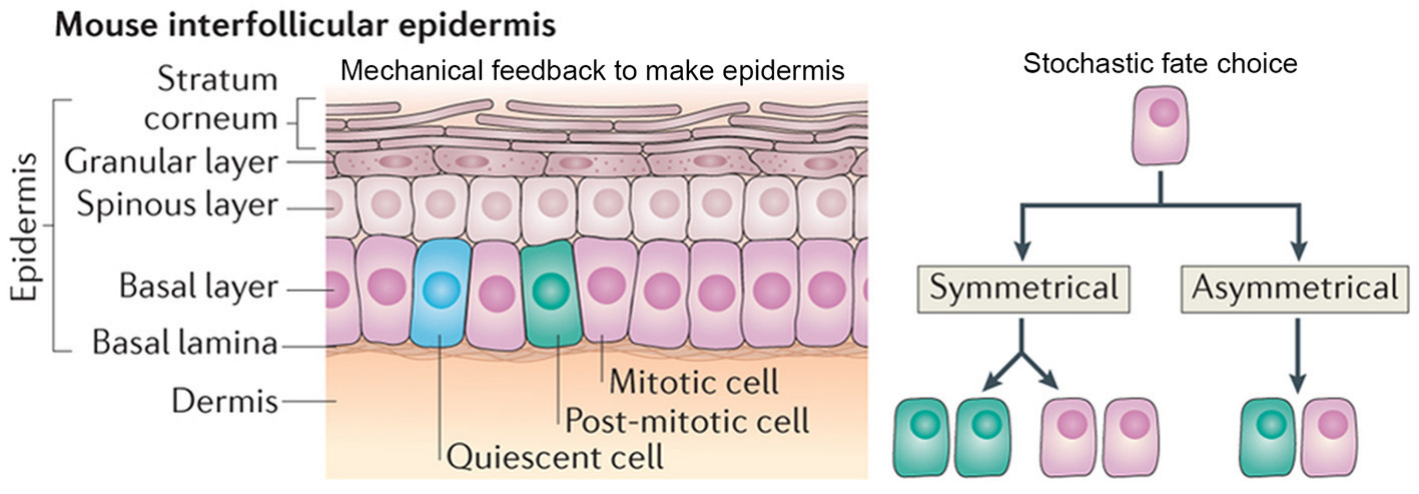
**Stochasticity promotes tissue reproducibility in mouse ear epidermis**. In the mouse interfollicular epidermis, regular columns of keratinocytes in the stratum corneum differentiate from cells in an underlying basal layer. A new model suggests that the daughters of mitotically dividing basal cells within the basal layer adopt three potential fates in a stochastic but probabilistic manner: (1) two mitotically dividing basal cells, (2) two post-mitotic differentiating basal cells, or (3) one mitotically dividing basal cell and one post-mitotic differentiating basal cell. Post-mitotic cells differentiate into flattened hexagonal keratinocytes and the mechanical properties of the cell shape ensure the regularity of the stratum corneum. Reprinted with permission from MacMillan Publishers Ltd: Solanas and Benitah (2013).

The cellular arrangement of the interfollicular epidermis is another example of how regular patterns can arise via stochastic processes. In the mouse ear, the epidermis is arranged into columnar structures of hexagonal keratinocytes. Keratinocytes differentiate from the underlying cells, which are produced by the proliferative cells in the basal cell layer (Figure 8; Solanas and Benitah, 2013).

It has long been hypothesized that the interfollicular epidermis is organized into “epidermal proliferative units.” The epidermal proliferative units hypothesis assumes that underlying the center of each keratinocyte column, there is a slow-cycling stem cell in the basal layer, which will give rise to transient amplifying daughter cells. These daughter cells will then eventually differentiate into keratinocytes (Allen and Potten, 1974; Potten, 1981). This model assumes that epidermal proliferative units are spatially constrained because each keratinocyte must be supported by an underlying basal cell.

Recently, a new model—which challenges the epidermal proliferative unit model—maintains epidermal homeostasis without use of a slow-cycling stem cell. This model suggests that all cells in the basal layer are equivalent and can give rise to daughters with three random cell fates: (1) two proliferating basal cells, (2) two cells differentiating into keratinocytes, or (3) one proliferating basal and one differentiating keratinocyte (Figure 8; Clayton et al., 2007). In this model, each fate exists with equal probability, creating a balance between cells undergoing proliferation and differentiation. One of the key predictions of this model is that the spatial arrangement of basal cells is random and doesn't correlate with that of the keratinocytes above it (Clayton et al., 2007).

Doupé et al. (2010) investigated whether the spatial arrangement of mouse ear epidermal cells was random. Using quantitative genetic cell lineage tracing and 3D imaging, the authors saw that basal cells often crossed the columnar boundaries and gave rise to the three proposed random cell fates. Additionally, by looking at the time distribution of the cell cycle, the authors showed that entry into cell cycle varied stochastically. These results overall imply that tissue organization within the mouse ear epidermis follows the stochastic model. Thus, if basal cell fate and division is random, how is tissue reproducibility achieved?

The authors propose that the morphological and physical constraints caused by the flattened hexagonal shape of the keratinocytes causes them to pack into regular columns, thus filtering out the stochasticity in the underlying basal cells (Figure 8). Furthermore, the authors suggest that undifferentiated basal cells may be regulated through contact inhibition and only divide when a cell leaves the layer through differentiating as a keratinocyte. These constraints allow for the generation of new keratinocytes without the presence of slow-cycling stem cells or transient amplifying cells. This model is very attractive for maintaining epidermal tissue homeostasis and easily explains how the epidermis is able to recover after injury. In several other biological systems, mechanical constraints also ensure tissue regularity despite stochasticity of cellular actions (Martin et al., 2009; Pouille et al., 2009; Marinari et al., 2012).

Mechanical feedback loops can also amplify stochastic differences between cells to promote organogenesis in the plant shoot apical meristem. The shoot apical meristem is an organized proliferating structure that generates all the aerial plant tissues and organs. The SAM is made up of three zones: (1) the central zone, which consists of a small population of stem cells needed to maintain the meristem, (2) the surrounding peripheral zone, where primordia grow to give rise to successive organs, and (3) the rib meristem, which gives rise to the stem (Murray et al., 2012). In the meristem, the cells generally start out isotropic but transition into being anisotropic as the primordia emerge. Cortical microtubule dynamics have been shown to play an important role in facilitating this transition. The cortical microtubules orient along the principal direction of tensile stress in cells (Hamant et al., 2008; Sampathkumar et al., 2014). Cellulose synthase complexes track along the cortical microtubules to deposit new cellulose microfibrils in the cell wall (Paredez et al., 2006), which reinforce the wall to resist stress and orient growth.

Uyttewaal et al. (2012), used a cell-based 2D model to show that a cell's response to mechanical stress (mediated by cortical microtubules) influences growth homeostasis by modulating intercellular growth variability. The Uyttewaal et al. model is based upon four major assumptions: (1) each cell has its own intended growth rate; however, the actual growth rate of one cell must partially compensate for the intended growth rates of its neighboring cells; (2) because cell activity is inherently noisy, each cell's intended growth rate is subjected to stochastic fluctuations causing cellular heterogeneity amongst a population of cells; (3) cell growth is symplastic; cells do not migrate or detach from one another; (4) differential growth rates between adjacent cells generate a local pattern of mechanical stress, triggering cortical microtubules and cellulose microfibrils to reorient themselves to promote local anisotropic growth.

**Figure 9:**
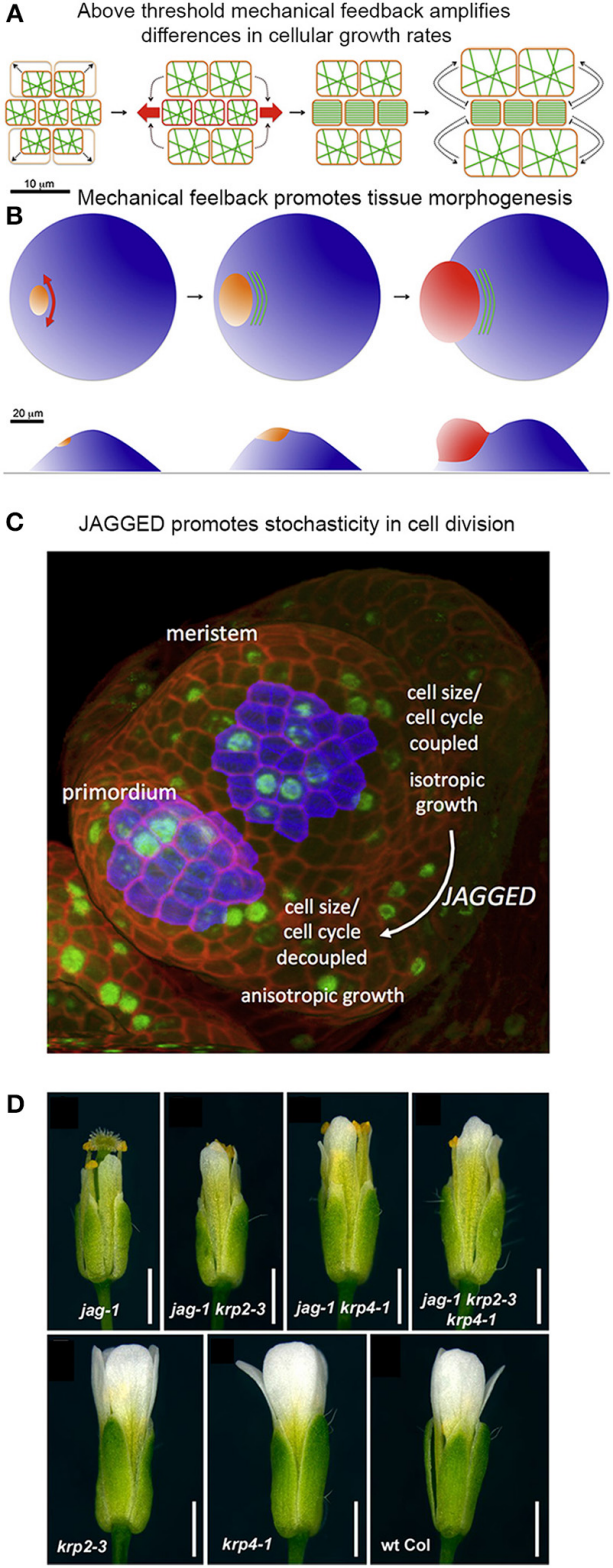
**Mechanical stress and genetics can enhance stochasticity to promote heterogeneous growth. (A,B)** Mechanical feedback loops can amplify variability in cellular growth rates to direct morphogenesis. **(A)** Microtubule (green) orientation is initially random when little mechanical force is exerted on cells. If some cells grow more rapidly than their neighbors (top and bottom cells in the left panel), they exert a force on the slower growing cells (in the middle). Once force surpasses a particular threshold (red arrows), microtubules will re-orient in a parallel manner to induce anisotropic growth that resists the mechanical stress. This further amplifies differential growth between neighboring cells. **(B)** On the organ scale, mechanical stress can amplify differences in growth rates to promote tissue morphogenesis. The shoot apical meristem (blue) endures increased mechanical stress and anisotropic growth in the peripheral zone, where the organ primordium is emerging (orange to red). Cortical microtubules (green) orient parallel to the emerging organ. Reprinted from Uyttewaal et al. (2012), Copyright, with permission from Elsevier. **(C)** JAGGED promotes variability by uncoupling cell size from the cell cycle in the organ primordia. In the floral meristem, cells grow isotropically and must reach a certain size to divide. This figure was reproduced from Schiessl et al. (2012), Copyright Elsevier. **(D)** JAGGED represses expression of the cyclin-dependent kinase inhibitors *KRP2* and *KRP4* to promote variability. Double and triple mutants with *jag-1, krp4-1*, and *krp2-3* partially rescue the *jag-1* phenotype, suggesting that JAG initiates cellular variability partially through repression of KRPs. Reprinted from Schiessl et al. (2014).

Through testing different growth scenarios in the model, the authors observed *in silico* that increasing the ability of a cell to respond to mechanical stresses (i.e., those caused by differential growth) decreases the potential for a population of cells to have variable growth rates, thus promoting homogeneous cell growth. In other words, contiguous cells with distinct intended growth rates can reach a growth compromise via mechanical interactions, thus buffering stochasticity in the growth rate. However, if the mechanical stress feedback surpasses a particular threshold, cells switch from having a homogenized growth rate to a highly variable growth rate (Figure 9A). As cells respond strongly to growth-derived stresses, the rapid reorientation of their microtubules leads to a modification of their anisotropic growth, which further increases the growth heterogeneity between adjacent cells in a positive feedback loop, thus amplifying stochastic differences to promote differences between cells. Overall, this model predicts that local mechanical stresses—induced by differential growth—can either reduce or promote growth heterogeneity.

Analysis of growth in real meristems rather supports the latter scenario—that mechanical stress feedback amplifies stochastic differences between cell growth rates. For example, regions where stress levels are the most directional (e.g., the boundary between the meristem and an emerging organ) are also the regions where growth is the most heterogeneous (Figure 9B). Conversely, reducing the ability for meristematic cells to respond to mechanical stress in the microtubule-severing mutant *katanin* results in more homogeneous growth. Remarkably, stochasticity, in relation to mechanical stress, acts as an instructing signal that actively maintains a level of variability. Such high variability may promote cells to undergo differential growth rates at a lower cost and thus potentiate organogenesis (Figure 9B; Uyttewaal et al., 2012). Further work is required to analyze whether a similar scenario is occurring in other plant and animal contexts.

### A transcription factor promotes stochasticity in plant cell division

Genetic mechanisms can also enhance stochasticity to promote regularity in organogenesis. Schiessl et al. (2012) demonstrated that the transcription factor JAGGED promotes stochasticity in the cell division and isotropic growth in initiating sepal primordia on the flanks of the floral meristem. Schiessl et al. performed dynamic 3D analysis of cell geometry, growth and DNA synthesis of wild type and *jagged-1* mutant sepal primordia and compared it to the isotropically growing floral meristem. In this study, Scheissl et al. found that without JAGGED, the sepal primordium loses its ability to grow anisotropically, resembling the floral meristem. Additionally, Schiessl et al. discovered that in both the floral meristem and *jagged-1* sepal primordia, cell cycle and cell volume are linked, where a certain cell volume must be attained to initiate entry into S phase of the cell cycle. Whereas in wild-type sepal primordia, no correlation is found between S phase and cell volume (Figure 9C). This suggests that JAGGED is needed to uncouple the cell cycle from cell volume, which overall leads substantial variability in sepal cells.

Interestingly, mutations in *JAGGED* have no major influence on sepal primordia emergence. This suggests that cellular heterogeneity is not a prerequisite for initial organ outgrowth in the flower. However, *jagged-1* mutants generate shorter and narrower petals and sepals. This raises the intriguing possibility that cell heterogeneity may be important for proper organ growth and morphology.

In 2014, Schiessl et al. performed Chip-Seq to identify gene targets of JAGGED. In their analysis, they found that JAGGED repressed various genes, including cyclin-dependent kinase inhibitors KRP2 and KRP4, which have previously been shown to control G1 to S-phase transitions during the cell cycle. This finding supports the argument that JAGGED induces cell proliferation during floral development. Furthermore, Schiessl et al. created double and triple mutants with *jagged-1, krp4-1 and krp2-3*. Remarkably, these mutants resulted in a partial rescue of the *jagged-1* phenotype (Figure 9D). Thus, JAGGED promotes cellular variability—partially through repression of KRPs—and the regular growth of the floral organs. We have a lot more to learn about how this cellular heterogeneity contributes to the regularity of organ growth.

## Conclusion and perspectives

It is not always easy to recognize stochasticity because it is often underlies regulated biological events. Nevertheless, many mechanisms take advantage of stochasticity to initiate important decisions necessary for proper development (Figure 1). Stochasticity is critical for creating small differences between identical neighboring cells. This does not suggest that deterministic regulation is not important during development. On the contrary, genetic and mechanical feedback loops are essential for amplifying this noise to solidify developmental decisions as well as for suppressing excess noise that could lead to deleterious developmental outcomes. These feedback loops are often instrumental in inducing patterning mechanisms that ensure regular development of the tissue. Thus, regular development often requires stochasticity to initiate the process.

As the role of stochasticity during multicellular development becomes more apparent, it is necessary to develop new quantitative technology to study it, notably to assess and compare averages together with standard deviations. In unicellular systems, the development of single-cell fluorescence assays has led to the rapid progression of knowledge about stochasticity in gene regulation. However, in multicellular systems, we need to create new techniques to effectively study stochasticity.

Studying stochasticity is challenging because it is a behavior, not a gene function, and therefore you cannot simply knock out or overexpress it to analyze its function. Thus, techniques that abate or enhance stochastic behavior must be developed to study the consequences of stochasticity during development. In a recent paper from Dar et al. (2014), noise-modulating compounds (e.g., chromatin remodeling factors) were successfully used to enhance or reduce noise. Therefore, using noise-modulating compounds to manipulate stochasticity seems promising for studying the role of stochasticity in asymmetric cell response and should be explored in other biological systems.

### Conflict of interest statement

The authors declare that the research was conducted in the absence of any commercial or financial relationships that could be construed as a potential conflict of interest.

## Footnotes

^1^TheFreeDictionary, *TheFreeDictionary.com*. Farlex, Inc. Available online at: http://www.thefreedictionary.com/stochasticity (Accessed May 26, 2014).
